# Contralateral Facial Innervation in Healthy Subjects and in Patients with Peripheral Facial Palsy

**DOI:** 10.3390/jcm13071846

**Published:** 2024-03-23

**Authors:** Halil Güllüoğlu, Hasan Armağan Uysal, Burhanettin Uludağ

**Affiliations:** 1 Department of Neurology, School of Medicine, Izmir Economy University, Medical Point Hospital, 35550 Izmir, Turkey; druysalarmagan@yahoo.com; 2 Department of Neurology, School of Medicine, Ege University, 35030 Izmir, Turkey; burhanettinuludag@gmail.com

**Keywords:** facial palsy, innervation, contralateral, glucocorticoid, physical therapy

## Abstract

**Background**: We aimed to investigate the extent of the response of the orbicularis oris muscle to stimulation of the contralateral facial nerve both in patients with peripheral facial palsy (PFP) and in healthy subjects. **Methods**: EMG was performed at 2–6 weeks after the onset of PFP in the patient group and at any time in the healthy control group. We performed nerve conduction testing, electroneurography, and surface and needle EMG. **Results**: A total of 276 participants (patients/healthy controls: 218/58) were analyzed. The extent of the response of the contralateral orbicularis oris muscles to facial nerve stimulation was higher in healthy controls compared to that in the affected group. The response of the contralateral orbicularis oris muscles to stimulation of the paralyzed facial nerve was more extensive in those patients to whom glucocorticoid or physical therapy had been given. Cross-facial innervation in the orbicularis oris muscle extended up to 1.5 cm in one-third of healthy controls and was higher than that in those with PFP. Glucocorticoid or physical therapy seemed to improve cross-innervation in facial palsy. **Conclusions**: Our findings suggest that the stimulus leading to the contralateral muscular response is mediated through crossing axons rather than muscular fibers.

## 1. Introduction

Peripheral facial palsy (PFP) is one of the most frequent neural injuries, and Bell’s palsy is the most common cause of unilateral facial palsy [1]. Bell’s palsy is an acute lower motor neuron paralysis which resolves in the majority of cases [1]. Although the etiological factor is unknown, viral, ischemic, auto-immune, or inflammatory factors could be possible etiologies. The condition is more common in people with diabetes [1,2].

Electromyography (EMG) provides an evaluation of facial nerve function and records the excitability of the muscles innervated by the facial nerve. At about 10 days after the onset of paralysis, compound muscle action potential (CMAP) in response to ipsilateral facial nerve stimulation reflects the loss of axonal function in the affected side [1,2]. Needle EMG measures denervation intensity, and hence the degree of axonal damage, 2 weeks after the onset of paralysis [1]. Axonal regeneration and signs of reinnervation from the ipsilateral nerve may be first observed within 3 months after the onset of paralysis [1,2].

In complete denervation of the facial muscles, small-amplitude motor unit action potentials (MUAPs) which appear in the orbicularis oris muscle may be activated by the stimulation of the contralateral facial nerve approximately 1 month after PFP onset [1]. Similar findings were also reported in other studies [3,4]. However, in some reports, it was not clear whether the activity was evoked by the stimulation of an unaffected facial nerve or whether it was the result of voluntary effort due to neural conduction or muscle fibers crossing the midline [2,4]. Moreover, in a previous study, facial nerve stimulation was shown not to lead to any response in the contralateral orbicularis oris muscle in healthy subjects [2].

Our objective was to examine whether contralateral facial nerve stimulation could elicit a response spanning from the midline to the lateral areas of the orbicularis oris muscle. We conducted this analysis in both healthy individuals and patients with peripheral facial paralysis (PFP) before the onset of the regeneration process.

## 2. Materials and Methods

### 2.1. Study Design

This retrospective study was conducted in the Neurology Department of İzmir University of Economics Medicalpoint Hospital and approved by the local Ethics Committee of İzmir Bakırçay University (decision no: 1102, research no: 1082, date: 12 July 2023). This study was conducted in accordance with the ethical standards laid down in the 1964 Declaration of Helsinki and its later amendments. Written informed consent was obtained from all of the participants.

### 2.2. Study Population

Adult patients who had been admitted with a diagnosis of PFP to the Neurology Clinics of İzmir University of Economics Medicalpoint Training and Research Hospital between 1 January 2019 and 1 July 2023 were included retrospectively. The diagnosis of PFP was based on clinical history and physical examination findings which were typical for the diagnosis. Those for whom data were missing and those whose signs and symptoms were attributed to a diagnosis other than PFP were not included in this study.

Healthy controls were selected from among adult individuals without PFP or apparent neurological disorder for whom data were available. Exclusion criteria for both groups consisted of a previous diagnosis of movement disorder, a previous history of brain surgery, a neurological disorder or cerebrovascular accident, previous treatment with botulinum toxin, treatment with central nervous system suppressants, or the presence of comprehension problems.

### 2.3. Data Collection

Demographic parameters (age and sex) and clinical variables (the affected side of facial palsy, co-existent illnesses, physical therapy, and glucocorticoid use in the 1st week) as well as EMG findings were recorded by using electronic and written patient files. Glucocorticoid use was defined as oral methylprednisolone treatment initiated at the onset of facial palsy at a dose of 1 mg/kg, which was gradually tapered and ceased on 10th day of palsy. Physical therapy was defined as the application of any physical therapy method continuously in the first 14 days of onset of facial palsy.

Based on the clinical findings, we used the House–Brackmann Facial Nerve Grading System to describe the degree of facial nerve dysfunction [5].

### 2.4. Electromyography Recordings

Electrophysiological studies were conducted using a Nihon Kohden (Tokyo, Japan) Electromyograph measuring system (Model: MEB-9400K). All study participants underwent electroneuromyography (EMG) performed by a single physiatrist. Distal motor latencies and amplitude were calculated using disc surface cup (Ag/AgCl) recording electrodes, which were 5 mm in diameter, and concentric needle EMG electrodes. To preclude artifacts, for each patient, the filter used was 500 Hz–10 kHz, the stimulation rate applied was 1 Hz, the duration was 0.3 ms, and the intensity was 35–40 mA. During surface electrode recording, stimulation was applied at the point anterior to the tragus (anterior to the stylomastoid foramen).

During orbicularis oculi muscle recording, an active electrode was placed at the inferior division of the orbicularis oculi muscle and a reference electrode at the superior division of the muscle. During the recording of alaeque nasi muscle, an active electrode was placed in the middle of the ipsilateral alaeque nasi muscle and a reference electrode at the conjunction of the nasal bone and the frontal bone. During surface electrode recording of the orbicularis oris muscle, an active surface electrode was placed 2 cm medial to midline in the upper division of the ipsilateral orbicularis oris muscle and a reference electrode at the conjunction of the nasal bone and the frontal bone. 

EMG was performed at 2–6 weeks after the onset of PFP in the patient group and at any time in the healthy control group.

Nerve conduction testing and electroneurography were performed to analyze compound muscle action potential in the right and left orbicularis oculi as well as the alaeque nasi muscles’ response to supramaximal electrical stimulation of the facial nerve. Surface EMG was performed using surface disc electrodes to evaluate the response of the right and left orbicularis oris muscles to electrical stimulation of the facial nerve at a point inferior to the tragus.

**Figure 1:**
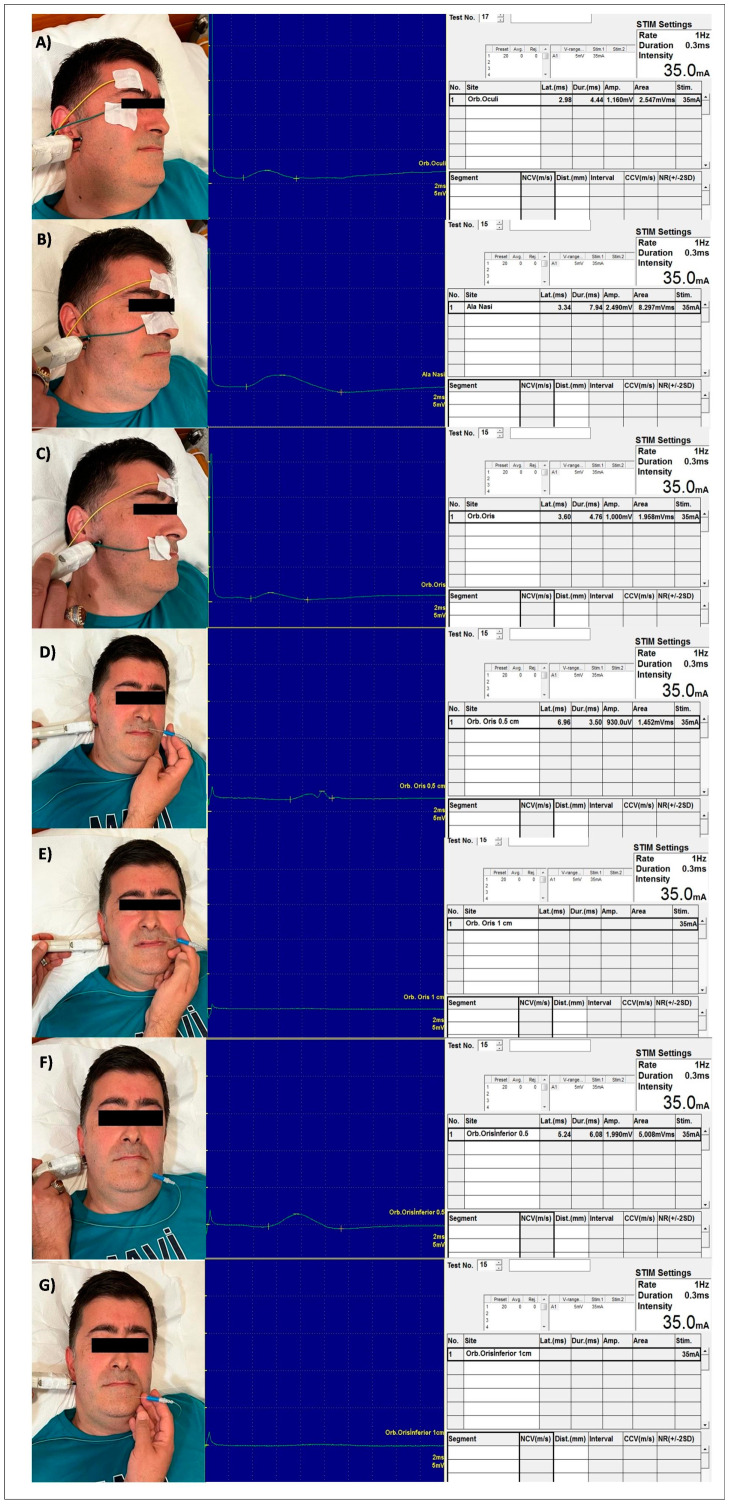
EMG analysis in response to stimulation of right facial nerve in a 46-year-old male patient. House–Brackmann score: 3; glucocorticoid was used in the 1st week; physical therapy was not given. (**A**) Orbicularis oculi recording by surface disc electrode, (**B**) alaeque nasi recording by surface disc electrode, (**C**) orbicularis oris recording from 2 cm medial to the midline by surface disc electrode, (**D**) orbicularis oris left division superior segment, recording from 0.5 cm lateral to the midline by concentric needle electrode, (**E**) orbicularis oris left division superior segment, recording from 1 cm lateral to the midline by concentric needle electrode, (**F**) orbicularis oris left division inferior segment, recording from 0.5 cm lateral to the midline by concentric needle electrode, (**G**) orbicularis oris left division inferior segment, recording from 1 cm lateral to the midline by concentric needle electrode.

Needle EMG using concentric needle electrodes was performed to analyze MUAPs from the orbicularis oculi, alaeque nasi, and orbicularis oris muscles. Needles were sequentially inserted at 0.5, 1, 1.5, 2, and 2.5 cm lateral to the midline in 4 different regions of the orbicularis oris muscle (left upper, right upper, left lower, and right lower). For each insertion site of the needle, the right and left facial nerves were respectively electrically stimulated at a point inferior to tragus. Latencies were measured in miliseconds (ms) and amplitudes in microvolts (µV). The EMG analysis of a participant is demonstrated in Figure 1.

### 2.5. Patient Groups

We grouped the participants into 2 groups: patients with PFP and healthy controls without facial palsy or any clinical illnesses. The patients were treated according to recent guidelines [6,7].

### 2.6. Statistical Analysis

The data obtained in this study were statistically analyzed using SPSS 25.0 software (IBM Corporation, Armonk, NY, USA) and Medcalc 14 (Acacialaan 22, B-8400 Ostend, Belgium). The conformity of the data to normal distribution was evaluated using the Shapiro–Wilk–Francia test. Homogeneity of variance was evaluated by the Levene test. When comparing two independent groups according to qualitative variables, the Mann–Whitney U test with Monte Carlo results was used. When comparing categorical variables to each other, the Pearson chi-square and the Fisher–Freeman–Halton test with the Monte Carlo simulation technique were used. When comparing quantitative variables to each other, Spearman’s rho correlation was used. To detect the relationship between the real classification of the procedure’s success and the classification made by the cut-off values, sensitivity and specificity ratios as well as positive and negative predictive values were expressed by ROC (receiver operating characteristic) curve analysis. Quantitative variables are presented as means (standard deviation) and medians (minimum–maximum) and categorical variables as number (n) and percentage (%) in the tables. Variables were evaluated at a 95% confidence level and a value of *p* ≤ 0.05 was accepted as statistically significant.

## 3. Results

A total of 276 participants were analyzed. The mean age was 35.65 (±7.45) and the male/female ratio was 85/191. The number of patients with facial palsy was 218. The median duration between the onset of palsy and EMG was 29 days (17–40), with glucocorticoids used in the first week of facial palsy in 176 patients.

Responses were recorded by surface EMG electrodes from 2 cm lateral to the midline for both the right and left orbicularis oris muscles in 165 patients with PFP and in all healthy controls (Supplement Table S2).

In patients with PFP and in healthy controls, no response was obtained from the left orbicularis oculi or left alaeque nasi muscles upon the stimulation of the right facial nerve. In both patients with PFP and in healthy controls, no response was obtained from the right orbicularis oculi or right alaeque nasi muscles upon stimulation of the left facial nerve (not shown in the tables).

Cross-facial nerve stimulation did create a response up to 1.5 cm lateral to the midline in contralateral upper left, upper right, and lower left orbicularis oris muscles in 34.48% of the participants in the healthy control group, but did not elicit any response beyond 1.5 cm. Cross-facial nerve stimulation did create a response up to 1 cm lateral to the midline in the contralateral lower right orbicularis oris muscle in 51.72%, in the contralateral lower left orbicularis oris muscle in 86.21%, in the contralateral upper right orbicularis oris muscle in 86.21%, and in the contralateral upper left orbicularis oris muscle in 68.97% of the participants in the healthy control group (Supplement Table S3).

When paralyzed facial nerves were stimulated in the patient group and the mean value of both right and left facial nerve stimulation was evaluated in the healthy control group, the responses of the contralateral upper and lower orbicularis oris muscles were extended to a greater degree laterally from the midline in the healthy controls. When unaffected facial nerves were stimulated in the patient group and the mean value of both right and left facial nerve stimulation was evaluated in the healthy control group, the response of the contralateral upper and lower orbicularis oris muscles was extended to a greater degree laterally from the midline in the healthy controls when compared to that seen in the patient group (Table 1).

The response of the contralateral upper and lower orbicularis oris muscles to the stimulation of paralyzed facial nerves in the patient group was extended to a greater degree in those in whom glucocorticoids had been used in the first week of palsy and in those to whom physical therapy had been given (Table 2).

The House–Brackmann score was negatively correlated with the extension of the response of the contralateral upper and lower orbicularis oris muscles (on the paralyzed side) to the stimulation of the unaffected facial nerves and positively correlated with the extension of the response of the contralateral upper orbicularis oris muscle on the unaffected side to the stimulation of the paralyzed facial nerves (Table 3).

**Figure 2:**
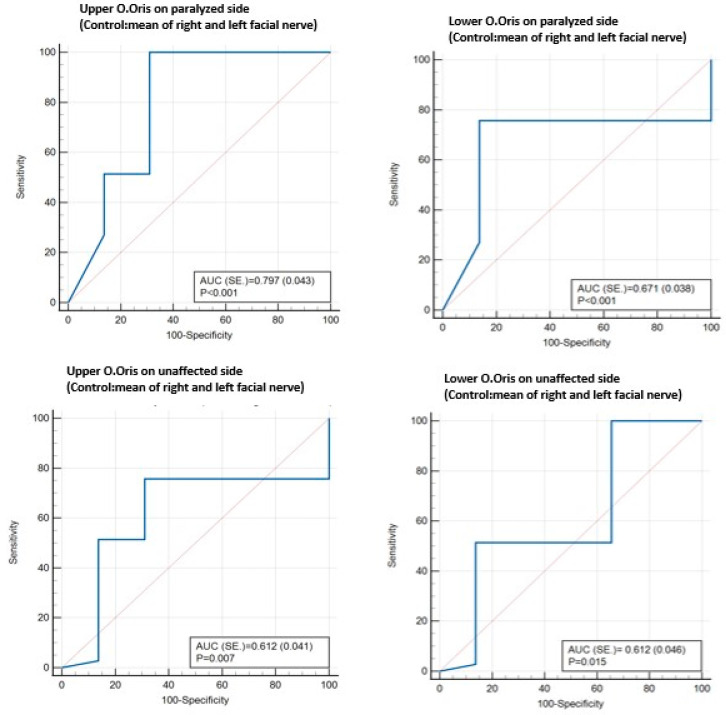
Extension of response of orbicularis oris muscles beyond midline to stimulation of contralateral facial nerve.

ROC curve analysis showed the cut-off values for age, House–Brackmann score, and the extension of the response of the orbicularis oris muscles beyond the midline to the stimulation of the contralateral facial nerve (Table 4 and Figure 2).

The electroneurography findings and the denervation, reinnervation, recruitment, and interference patterns are set out in Supplement Tables S1 and S4.

## 4. Discussion

We showed that cross-facial innervation in the orbicularis oris muscle extends up to 1 cm lateral from the midline in the majority of healthy controls and up to 1.5 cm in one-third of the same. Cross-facial innervation was lesser in patients with peripheral facial palsy in response to both unaffected and paralyzed facial nerves compared to that seen in healthy controls. Cross-facial innervation was not observed for orbicularis oculi or alaeque nasi muscles either in the PFP or healthy control group.

Our findings suggest that stimulation of the contralateral facial nerve triggered a response in the orbicularis oris muscle up to 1–1.5 cm lateral from the midline in healthy controls. Casanova-Molla et al. found no response to contralateral facial nerve innervation in the orbicularis oris muscle in healthy subjects [2]. Based on this, they proposed that crossing muscle fibers rather than cross-innervation might conduct impulses in facial palsy. The procedure to confirm this is as follows. A reference surface electrode is placed on a myoelectrically inactive area of the body, such as the manubrium sterni. Bipolar concentric needle electrodes provide the most suitable area for analyzing MUAP waveforms. The selection of facial muscles to be examined depends on both the identification of anatomical reference points and the clinical information required. If the entire facial nerve is to be anatomically examined, muscles such as the frontalis, orbicularis oculi, orbicularis oris, and zygomaticus are chosen. These muscles can be easily identified through mimic movements. The evaluation of contralateral stimulation is also indirectly performed in this manner. However, in our study, nerve conduction findings obtained during contralateral facial nerve stimulation in healthy subjects, such as latencies and amplitudes, did point to a neural conduction. Hence, we suggest that response maintenance in the contralateral orbicularis oris muscle is by cross-innervation rather than crossing muscular conduction. In an important previous study undertaken by Gambi and Tonali, stimulation of the facial nerve elicited a response in both the contralateral superior and inferior orbicularis oris muscles at up to 2.5 cm distance from the midline in four healthy subjects [8]. They showed that there was a more extensive response, up to 3 cm from the midline, to contralateral stimulation of the unaffected nerve in the orbicularis oris muscle in all patients with facial palsy. According to that study, the extent of the contralateral response upon stimulation of unaffected facial nerves was higher, at least to some degree, in facial palsy compared to that seen in healthy subjects. The motor endplates of facial muscles are located in the immediate vicinity of nerve entry points and are positioned eccentrically. This differs from skeletal muscles, where motor endplates form a narrow band stretching across the central zone. This may explain why the phenomenon of contralateral reinnervation is likely observed only in facial muscles close to the midline. Reinnervation by the ipsilateral trigeminal nerve and terminal branches of the mandibular nerve has also been reported in humans. However, Passerini et al. obtained no muscle response beyond the midline, suggesting that facial nerve innervation was limited to one side only [9].

The extent of contralateral innervation has been less investigated. In one study, it was proposed that acute-onset paralysis was associated with limited reinnervation, but contrasting findings have also been reported [9,10]. Disease duration was also shown not to be associated with reinnervation pattern [9]. However, in another study, contralateral reinnervation of facial muscles was present in all patients with facial palsy for at least 9 months but not in healthy subjects [11]. As contralateral cross-innervation was absent in healthy subjects, the change in facial palsy was deemed to be evidence of contralateral reinnervation. We showed that glucocorticoid use in the first week of paralysis or the administration of physical therapy in patients with PFP might improve the extent of the response of the orbicularis oris muscle on the unaffected side to stimulation of the paralyzed facial nerve. Glucocorticoid use or physical therapy did not improve the response of the orbicularis oris muscle on the paralyzed side to stimulation of the unaffected facial nerve. In a previous study, the utilization of glucocorticoids was found to influence the likelihood of achieving full recovery in cases of facial palsy [12]. The possible mechanisms associated with the benefit of glucocorticoid use in facial palsy may consist in the regulation of the immune response acting in the pathogenesis of facial palsy or a direct reduction in perineural edema, axonal degeneration, or lipid peroxidation [12,13]. The effectiveness of physical therapy in enhancing recovery among individuals with peripheral facial paralysis has also been demonstrated, particularly when administered during the early stages of the condition [14,15].

We showed that stimulation of the unaffected facial nerve did elicit a response in the contralateral orbicularis oris muscle, albeit to a lesser extent in the patient group than contralateral innervation in healthy subjects. Reinnervation in the paralyzed facial nerve has been shown to occur at about the third month of palsy [1,2]. We evaluated patients who were approximately between the second and the sixth week of palsy. As a result of the specific timeframe during which our measurements were conducted, our assessment focused on contralateral cross-innervation within the patient group, as opposed to ipsilateral reinnervation. However, Gilhuis et al. showed that contralateral reinnervation might be observed 14 days after the onset of facial palsy [11]. Passerini et al. reported a similar finding for contralateral reinnervation as early as the 16th day after the onset of facial palsy [9]. Gilhuis et al. also showed that contralateral reinnervation occurred together with ipsilateral reinnervation in facial palsy [11]. It may be proposed that our assessments consist of both ipsilateral reinnervation of the paralyzed facial nerve and contralateral innervation of the unaffected facial nerve in patients with PFP. Deciphering these variations might pose a challenge.

Nonetheless, owing to the observed reduced response of the contralateral orbicularis oris muscles to both the unaffected and paralyzed facial nerves in patients as compared to healthy controls, we suggest that peripheral facial paralysis (PFP) might have a more pronounced impact on the functionality of contralateral unaffected nerve fibers crossing over to the paralyzed side. It might be possible that this might result in structural or functional alterations of the muscle on the paralyzed side, which might lead to a decreased response to stimulation by the unaffected facial nerve.

We demonstrated the presence of cross-facial innervation in the orbicularis oris muscle in cases of peripheral facial palsy; however, this phenomenon exhibited a slightly diminished effectiveness when compared to healthy subjects. Contralateral reinnervation in facial palsy has been demonstrated in previous studies [2,4,10,11,16]. Perioral muscle fibers were shown to cross the midline [16,17]. In a postmortem study, it was shown that the fibers of the orbicularis oris muscle decussate in the midline [18]. In some studies, it was suggested that the action potential triggered by the stimulation of the contralateral facial nerve should be mediated by muscle fibers; nevertheless, previous reports have indicated that axons crossing the midline were indeed capable of innervating contralateral motor endplates [4,11,19,20]. Casanova-Molla et al. revealed a response to contralateral facial nerve stimulation in the orbicularis oris muscle in patients with complete facial palsy, but they did not observe cross-facial innervation in healthy subjects [2]. They also showed that conduction velocity, the refractory period, and latency variability in patients with facial palsy were associated with muscle fiber impulse propagation rather than conduction via the motor axon. The lack of response to contralateral facial nerve stimulation in healthy subjects also supported their findings, which did point to conduction via crossing muscle fibers rather than crossing nerve axons. Furthermore, the absence of latency changes over time in the facial palsy group, as observed in that study, could potentially be linked to conduction through muscle fibers [2]. In contrast, our findings suggested that cross-innervation was present in healthy subjects, as noted above.

Again, nerve conduction research conducted by our facial palsy group also indicated the occurrence of cross-innervation. Moreover, we showed that there was a difference in the extent of cross-conduction from the midline between the groups in favor of healthy subjects. This finding could also suggest that the nature of conduction is more aligned with cross-innervation rather than conduction through muscle fibers crossing the midline because crossing fibers of the muscle on the unaffected side would not be expected to be injured by contralateral facial paralysis and conduction via these fibers would not be expected to decrease in the transition from a healthy state to facial palsy [20]. Trojaborg et al. held that conduction crosses the midline via muscle fibers based on conduction velocities [21]. In other previous studies, cross-conduction via a neural connection was suggested; moreover, long latencies of responses were shown to be associated with unmyelinated, recently sprouted nerve branches [4].

Cross-innervation of the contralateral muscle might be suggested as a protective factor in the maintenance of muscle function, at least partially. However, it has been shown that innervation of the muscle fiber by an axon might limit the growth of other axons [22]. Therefore, contralateral reinnervation occurring after facial palsy might be an unfavorable process preventing ipsilateral paralyzed facial nerve reinnervation. We showed that cross-facial innervation was already present in healthy subjects and decreased in facial palsy. We did not perform a sequential analysis, which might have shown a temporary change in the process of reinnervation.

The orbicularis oris muscle was the most frequently studied muscle in the analysis of cross-facial nerve innervation in previous studies [2,4]. Various features of the orbicularis oris muscle, such as its anatomical features, its sphincter-like structure, round shape, or lack of fascia separating the muscular fibers, which consist of multiple motor endplates, were proposed to facilitate cross-conduction [18,23]. We also showed cross-facial innervation for the orbicularis oris muscle in healthy controls and to a lesser degree in PFP patients. Additionally, we analyzed cross-facial innervation in the orbicularis oculi and alaeque nasi muscles. However, we could not show any cross-innervation for the alaeque nasi and orbicularis oculi muscles. In a previous study, no response was recorded in the orbicularis oculi on the affected side upon ipsilateral stimulation or as a result of contralateral facial nerve stimulation [2].

However, contrasting findings have also been reported. Facial muscles other than the orbicularis oris, such as the nasalis and chin muscles, have also been shown to be innervated by the contralateral facial nerve [4,9,11,21]. The contralateral innervation of facial muscles other than the orbicularis oris remains to be elucidated.

We included a significant number of patients with peripheral facial palsy and performed nerve conduction studies, surface EMG, and needle EMG. The specific timeframe during which we conducted EMG assessments on patients with peripheral facial palsy allowed us to accurately evaluate contralateral facial innervation, rather than focusing solely on ipsilateral reinnervation. To our knowledge, the present study is the first study to show any benefit with respect to glucocorticoid or physical therapy on cross-innervation. We analyzed the orbicularis oris, orbicularis oculi, and alaeque nasi muscles. We could not perform a sequential analysis, which might have shown a temporary change in the process of contralateral and ipsilateral reinnervation.

Our study also has some limitations. It is possible that the use of medications affecting EMG conduction in patients diagnosed with PFP could yield various clinical outcomes. However, given the large number of patients and the similarities in age and clinical characteristics among them, these differences may have been negligible. Additionally, more frequent EMG applications during patient follow-ups could provide greater insight into the results, and therefore, updating the follow-up intervals with new studies could be beneficial.

## 5. Conclusions

We showed that cross-facial innervation in the orbicularis oris muscle extends up to 1 cm lateral from the midline in the majority of healthy controls. No cross-innervation was found for the orbicularis oculi or alaeque nasi muscles. Comparatively, the cross-facial innervation of the orbicularis oris muscle appears to be diminished in patients with peripheral facial palsy when contrasted with that in healthy subjects. However, the application of glucocorticoid treatment or physical therapy has the potential to enhance this phenomenon among those patients. Our findings suggest that facial nerve stimulation leading to a contralateral muscular response is conducted via crossing nerve fibers rather than crossing muscular fibers. We recommend temporary EMG assessments in patients with facial palsy.

## Figures and Tables

**Table 1:** Demographic, clinical, and EMG findings of the participants.

	Control	PFP	*p*
	(n = 58)	(n = 218)	
**Sex (male), _n (%)_**	**21 (36.2)**	64 (29.5)	0.340 ᶜ
**Age (year), _median (min/max)_**	34 (21/45)	38 (21/53)	**0.023 ᵘ**
**Side of PFP (left), _n (%)_**	0 (0)	108 (49.5)	-
**House–Brackmann score, _median (min/max)_**	1 (1/1)	4 (2/6)	**<0.001 ᵘ**
**Duration between PFP and EMG, _median (min/max)_**	-	29 (17/40)	-
**Glucocorticoid use in the 1st week of PFP (yes), _n (%)_**	0 (0)	176 (80.7)	-
**Co-existent illness, _n (%)_**			0.113 ᶠᶠ
Absent	36 (62.1)	97 (44.5)	
Rheumatoid arthritis	7 (12.1)	23 (10.6)	
Hashimoto thyroiditis	7 (12.1)	37 (17)	
Ankylosing spondylitis	0 (0)	5 (2.3)	
Type 2 diabetes	8 (13.8)	56 (25.7)	
**Physical therapy (yes), _n (%)_**	0 (0)	167 (76.6)	**<0.001 ᶜ**
**Response of O. Oris to stimulation of contralateral facial nerve**			
**Upper O. Oris on paralyzed side (control: right facial nerve), _median (min/max)_**	1 (0/1.5)	0.5 (0/1)	**<0.001 ᵘ**
**Upper O. Oris on paralyzed side (control: left facial nerve), _median (min/max)_**	1 (0/1.5)	0.5 (0/1)	**<0.001 ᵘ**
**Upper O. Oris on paralyzed side (control: mean of right and left facial nerve), _median (min/max)_**	1.25 (0/1.25)	0.5 (0/1)	**<0.001 ᵘ**
**Lower O. Oris on paralyzed side (control: right facial nerve), _median (min/max)_**	1 (0/1.5)	0.5 (0/1.5)	**<0.001 ᵘ**
**Lower O. Oris on paralyzed side (control: left facial nerve), _median (min/max)_**	1 (0/1)	0.5 (0/1.5)	**0.031 ᵘ**
**Lower O. Oris on paralyzed side (control: mean of right and left facial nerve), _median (min/max)_**	0.75 (0/1.25)	0.5 (0/1.5)	**<0.001 ᵘ**
**Upper O. Oris on unaffected side (control: right facial nerve), _median (min/max)_**	1 (0/1.5)	0.5 (0/1.5)	0.082 ᵘ
**Upper O. Oris on unaffected side (control: left facial nerve), _median (min/max)_**	1 (0/1.5)	0.5 (0/1.5)	**0.001 ᵘ**
**Upper O. Oris on unaffected side (control: mean of right and left facial nerve), _median (min/max)_**	1.25 (0/1.25)	0.5 (0/1.5)	**0.009 ᵘ**
**Lower O. Oris on unaffected side (control: right facial nerve), _median (min/max)_**	1 (0/1.5)	0.5 (0/1)	**<0.001 ᵘ**
**Lower O. Oris on unaffected side (control: left facial nerve), _median (min/max)_**	1 (0/1)	0.5 (0/1)	0.725 ᵘ
**Lower O. Oris on unaffected side (control: mean of right and left facial nerve), _median (min/max)_**	0.75 (0/1.25)	0.5 (0/1)	**0.006 ᵘ**

ᶜ Pearson chi square test (Monte Carlo); ᶠᶠ Fisher–Freeman–Halton test (Monte Carlo); ᵘ Mann–Whitney U test (Monte Carlo); PFP: peripheral facial palsy; min: minimum; max: maximum; O. Oris: orbicularis oris muscle.

**Table 2:** Association of response of orbicularis oris muscle to contralateral facial nerve stimulation with glucocorticoid use, co-existent illness, and physical therapy.

	Glucocorticoid Use in 1st Week		*p*	Co-Existent Illness		*p*	Physical Therapy		*p*
	Absent	Present		Absent	Present		Absent	Present	
	(n = 42)	(n = 176)		(n = 97)	(n = 121)		(n = 51)	(n = 167)	
**Upper O. Oris on paralyzed side, _median (min/max)_**	**0.5 (0/1)**	1 (0/1)	0.054	0 (0/1)	1 (0/1)	**<0.001**	0.5 (0/1)	1 (0/1)	0.225
**Lower O. Oris on paralyzed side, _median (min/max)_**	0.5 (0/1.5)	0.5 (0/1.5)	0.281	0 (0/1.5)	0.5 (0/1.5)	**<0.001**	0.5 (0/1.5)	0.5 (0/1.5)	0.084
**Upper O. Oris on unaffected side, _median (min/max)_**	0.5 (0/1.5)	1 (0/1.5)	**0.003**	1 (0/1.5)	0.5 (0/1.5)	0.988	0.5 (0/1.5)	1 (0/1.5)	**0.023**
**Lower O. Oris on unaffected side, _median (min/max)_**	0.5 (0/1)	1 (0/1)	**<0.001**	0.5 (0/1)	1 (0/1)	**<0.001**	0.5 (0/1)	1 (0/1)	**0.001**

Mann–Whitney U test (Monte Carlo); min: minimum; max: maximum; O. Oris: orbicularis oris muscle.

**Table 3:** Correlation of House–Brackmann score with response of O. Oris to contralateral facial nerve stimulation.

	House–Brackmann Score	
	r	*p*
**Upper O. Oris on paralyzed side**	−0.346	**<0.001**
**Lower O. Oris on paralyzed side**	−0.607	**<0.001**
**Upper O. Oris on unaffected side**	0.186	**0.006**
**Lower O. Oris on unaffected side**	−0.051	0.453

Spearman correlation test; r: correlation coefficient; O. Oris: orbicularis oris muscle.

**Table 4:** ROC curve analysis showing cut-off values of the factors associated with PFP.

Reference: PFP	Cut-Off	Sensitivity	Specificity	+PV	−PV	AUC ± SE	*p* Value
**Age**	>38	48.62	79.31	89.8	29.1	0.595 ± 0.04	**0.017**
**House–Brackmann score**	>1	100	100	100	100	1.000 ± 0.000	**<0.001**
**Upper O. Oris on paralyzed side (control: right facial nerve)**	≤1	100	34.48	85.2	100	0.692 ± 0.044	**<0.001**
**Upper O. Oris on paralyzed side (control: left facial nerve)**	≤0.5	51.38	86.21	93.3	32.1	0.755 ± 0.040	**<0.001**
**Upper O. Oris on paralyzed side (control: mean of right and left facial nerve)**	≤1	100	68.97	92.4	100	0.797 ± 0.043	**<0.001**
**Lower O. Oris on paralyzed side (control: right facial nerve)**	≤0.5	75.69	68.97	90.2	43	0.671 ± 0.040	**<0.001**
**Lower O. Oris on paralyzed side (control: left facial nerve)**	≤0.5	75.69	51.72	85.5	36.1	0.587 ± 0.039	**0.024**
**Lower O. Oris on paralyzed side (control: mean of right and left facial nerve)**	≤0.5	75.69	86.21	95.4	48.5	0.671 ± 0.038	**<0.001**
**Upper O. Oris on unaffected side (control: right facial nerve)**	≤0.5	51.38	68.97	86.2	27.4	0.571 ± 0.045	0.118
**Upper O. Oris on unaffected side (control: left facial nerve)**	≤0.5	51.38	86.21	93.3	32.1	0.633 ± 0.042	**0.001**
**Upper O. Oris on unaffected side (control: mean of right and left facial nerve)**	≤1	75.69	68.97	90.2	43	0.612 ± 0.041	**0.007**
**Lower O. Oris on unaffected side (control: right facial nerve)**	≤1	100	34.48	85.2	100	0.654 ± 0.050	**0.002**
**Lower O. Oris on unaffected side (control: mean of right and left facial nerve)**	≤0.5	51.38	86.21	93.3	32.1	0.612 ± 0.046	**0.015**

ROC (receiver operating curve) analysis (Honley and McNell—Youden index J); AUC: area under the ROC curve; SE: standard error; +PV: positive predictive value; −PV: negative predictive value; O. Oris: orbicularis oris muscle; PFP: peripheral facial palsy.

## Data Availability

Data are available from the corresponding author upon reasonable request.

## Supplementary Materials

The following supporting information can be downloaded at: https://www.mdpi.com/article/10.3390/jcm13071846/s1, Table S1: Compound muscle action potentials in orbicularis oculi and alaeque nasi muscles. Table S2: Response of O.Oris recorded in surface EMG at 2 cm lateral from the midline on the upper lip. Table S3: Response of O.Oris to stimulation of contralateral and ipsilateral facial nerve on needle EMG in control and patient groups. Table S4: Denervation, reinnervation, recruitment and interference pattern at all needle insertion sites.
