# Supplementary material for: Contralateral Facial Innervation in Healthy Subjects and in Patients with Peripheral Facial Palsy
Source: J Clin Med. 2024 Mar 23;13(7):1846. doi: 10.3390/jcm13071846 (PMC11012713; doi:10.3390/jcm13071846)
Supplement: Supplementary file 1 [file jcm-13-01846-s001.zip › jcm-2919079-supplementary.pdf]

SUPPLEMENTARY MATERIALS

Table S1. Compound muscle action potentials in orbicularis oculi and alaeque nasi muscles.

	**Controls (n = 58)**		**PFP (n = 218)**		**Total (n = 276)**	
			**n (%)**			
**Supramaximal Electrical Stimulus (mA)**						
No response	0 (0)		0 (0)		0 (0)	
Response	58 (100)		218 (100)		276 (100)	
	**mean (SD.)**	**median (min-Q1-Q3-max)**	**mean (SD.)**	**median (min-Q1-Q3-max)**	**mean (SD.)**	**median (min-Q1-Q3-max)**
	34.05 (6.59)	35 (25-30-40-45)	36.35 (7.75)	35 (20-30-45-45)	35.87 (7.57)	35 (20-30-45-45)
**Right Orbicularis Oculi (latency: msec)**						
No response	0 (0)		0 (0)		0 (0)	
Response	58 (100)		218 (100)		276 (100)	
	**mean (SD.)**	**median (min-Q1-Q3-max)**	**mean (SD.)**	**median (min-Q1-Q3-max)**	**mean (SD.)**	**median (min-Q1-Q3-max)**
	2.37 (0.48)	2.21 (1.75-2.1-2.5-3.43)	3.32 (0.99)	3.07 (1.85-2.35-3.92-4.91)	3.12 (0.99)	2.55 (1.75-2.25-3.82-4.91)
**Right Orbicularis Oculi (amplitude: µV)**						
No response	0 (0)		0 (0)		0 (0)	
Response	58 (100)		218 (100)		276 (100)	
	**mean (SD.)**	**median (min-Q1-Q3-max)**	**mean (SD.)**	**median (min-Q1-Q3-max)**	**mean (SD.)**	**median (min-Q1-Q3-max)**
	3.87 (0.84)	4.1 (2.23-3.69-4.53-4.87)	3.58 (1.44)	3.11 (0.25-2.21-4.85-5.99)	3.64 (1.34)	3.97 (0.25-2.3-4.53-5.99)
**Left Orbicularis Oculi (latency: msec)**						
No response	0 (0)		53 (24.3)		53 (19.2)	
Response	58 (100)		165 (75.7)		223 (80.8)	
	**mean (SD.)**	**median (min-Q1-Q3-max)**	**mean (SD.)**	**median (min-Q1-Q3-max)**	**mean (SD.)**	**median (min-Q1-Q3-max)**
	2.47 (0.55)	2.4 (1.71-2.03-2.6-3.6)	3.74 (0.68)	3.51 (2.1-3.25-4.34-4.94)	3.41 (0.85)	3.35 (1.71-3.1-3.85-4.94)
**Left Orbicularis Oculi (amplitude: µV)**						
No response	0 (0)		53 (24.3)		53 (19.2)	
Response	58 (100)		165 (75.7)		223 (80.8)	
	**mean (SD.)**	**median (min-Q1-Q3-max)**	**mean (SD.)**	**median (min-Q1-Q3-max)**	**mean (SD.)**	**median (min-Q1-Q3-max)**
	3.84 (0.63)	4.14 (2.46-3.74-4.25-4.69)	3.44 (1.71)	3.6 (0.18-1.41-5.34-5.95)	3.54 (1.51)	3.69 (0.18-2.56-4.51-5.95)
**Right Alaeque nasi muscle (latency: msec)**						
No response	0 (0)		0 (0)		0 (0)	
Response	58 (100)		218 (100)		276 (100)	
	**mean (SD.)**	**median (min-Q1-Q3-max)**	**mean (SD.)**	**median (min-Q1-Q3-max)**	**mean (SD.)**	**median (min-Q1-Q3-max)**

	2.41 (0.63)	2.32 (1.57-1.94-2.55-3.66)	3.48 (0.98)	3.19 (1.96-2.49-3.98-4.99)	3.25 (1.01)	2.89 (1.57-2.39-3.93-4.99)
**Right Alaeque nasi muscle (amplitude: μV)**						
No response	0 (0)		0 (0)		0 (0)	
Response	58 (100)		218 (100)		276 (100)	
	**mean (SD.)**	**median (min-Q1-Q3-max)**	**mean (SD.)**	**median (min-Q1-Q3-max)**	**mean (SD.)**	**median (min-Q1-Q3-max)**
	4.55 (1.72)	3.83 (2.4-3.55-6.72-6.98)	3.62 (2.47)	2.77 (0.35-1.69-3.89-7.89)	3.82 (2.36)	2.96 (0.35-2.45-6.72-7.89)
**Left Alaeque nasi muscle (latency: msec)**						
No response	0 (0)		53 (24.3)		53 (19.2)	
Response	58 (100)		165 (75.7)		223 (80.8)	
	**mean (SD.)**	**median (min-Q1-Q3-max)**	**mean (SD.)**	**median (min-Q1-Q3-max)**	**mean (SD.)**	**median (min-Q1-Q3-max)**
	2.53 (0.51)	2.58 (1.52-2.15-2.78-3.74)	3.25 (0.93)	3.15 (2.09-2.21-4.42-4.65)	3.06 (0.9)	3.11 (1.52-2.2-3.69-4.65)
**Left Alaque nasi muscle (amplitude: μV)**						
No response	0 (0)		53 (24.3)		53 (19.2)	
Response	58 (100)		165 (75.7)		223 (80.8)	
	**mean (SD.)**	**median (min-Q1-Q3-max)**	**mean (SD.)**	**median (min-Q1-Q3-max)**	**mean (SD.)**	**median (min-Q1-Q3-max)**
	4.15 (1.41)	3.67 (2.12-3.35-5.14-6.58)	3.86 (2.68)	3.65 (0.25-0.92-7.21-7.51)	3.94 (2.41)	3.65 (0.25-2.19-6.52-7.51)

SD.:Standard deviation, min:minimum, Q1: Percentile 25,Q3: Percentile 75, max: Maximum msec:millisecond μV:microvolt PFP:peripheral facial palsy mA:miliamper

Table S2. Response of O.Oris recorded in surface EMG at 2 cm lateral from the midline on the upper lip.

	**Controls (n = 58)**		**PFP (n = 218)**		**Total (n = 276)**	
			**n (%)**			
**Right O.Oris (latency: msec)**						
No response	0 (0)		53 (24.3)		0 (0)	
Response	58 (100)		165 (75.7)		276 (100)	
	**mean (SD.)**	**median (min-Q1-Q3-max)**	**mean (SD.)**	**median (min-Q1-Q3-max)**	**mean (SD.)**	**median (min-Q1-Q3-max)**
	3.84 (0.59)	3.75 (3.1-3.35-4.12-4.96)	4.37 (1.4)	4.58 (2.05-3.61-4.81-6.42)	4.26 (1.29)	4.55 (2.05-3.35-4.69-6.42)
**Right O.Oris (amplitude: μV)**						
No response	0 (0)		53 (24.3)		0 (0)	
Response	58 (100)		165 (75.7)		276 (100)	
	**mean (SD.)**	**median (min-Q1-Q3-max)**	**mean (SD.)**	**median (min-Q1-Q3-max)**	**mean (SD.)**	**median (min-Q1-Q3-max)**
	4.86 (1.22)	4.44 (3.21-4.21-5.85-6.85)	5.38 (1.99)	4.68 (0.19-3.75-5.98-8.95)	5.27 (1.87)	4.63 (0.19-3.85-5.97-8.95)
**Left O.Oris (latency: msec)**						
No response	0 (0)		53 (24.3)		53 (19.2)	

Response		58 (100)		165 (75.7)		223 (80.8)
	**mean (SD.)**	**median (min-Q1-Q3-max)**	**mean (SD.)**	**median (min-Q1-Q3-max)**	**mean (SD.)**	**median (min-Q1-Q3-max)**
	3.99 (0.51)	3.86 (3.28-3.58-4.56-4.97)	5.08 (1.15)	4.49 (2.85-4.22-6.45-6.99)	4.8 (1.13)	4.35 (2.85-4.13-4.93-6.99)
**Left O.Oris (amplitude: µV)**						
No response		0 (0)		53 (24.3)		53 (19.2)
Response		58 (100)		165 (75.7)		223 (80.8)
	**mean (SD.)**	**median (min-Q1-Q3-max)**	**mean (SD.)**	**median (min-Q1-Q3-max)**	**mean (SD.)**	**median (min-Q1-Q3-max)**
	5.5 (1.33)	4.75 (3.71-4.52-6.95-7.22)	3.66 (2.09)	4.76 (0.35-0.85-5.29-6.21)	4.14 (2.08)	4.76 (0.35-3.74-5.39-7.22)

SD.:Standard deviation, min:minimum,Q1: Percentile 25,Q3: Percentile 75, max: Maximum msec:millisecond µV:microvolt PFP:peripheral facial palsy

Table S3. Response of O.Oris to stimulation of contralateral and ipsilateral facial nerve on needle EMG in control and patient groups.

	**Control (n = 58)**		**PFP (n = 218)**		**Total (n = 276)**	
			**n (%)**			
**Response of upper left O.Oris to right facial nerve stimulation 0,5 cm lateral (latency: msec)**						
No response		8 (13.79)		6 (2.75)		14 (5.1)
Responsive		50 (86.21)		212 (97.25)		262 (94.9)
	**mean (SD.)**	**median (min-Q1-Q3-max)**	**mean (SD.)**	**median (min-Q1-Q3-max)**	**mean (SD.)**	**median (min-Q1-Q3-max)**
	3.94 (0.67)	3.89 (3.24-3.43-3.98-5.21)	4.5 (1.4)	4.73 (2.05-3.38-5.48-6.59)	4.39 (1.31)	4.67 (2.05-3.43-4.92-6.59)
**Response of upper left O.Oris to right facial nerve stimulation 0,5 cm lateral (amplitude: µV)**						
No response		8 (13.79)		6 (2.75)		14 (5.1)
Responsive		50 (86.21)		212 (97.25)		262 (94.9)
	**mean (SD.)**	**median (min-Q1-Q3-max)**	**mean (SD.)**	**median (min-Q1-Q3-max)**	**mean (SD.)**	**median (min-Q1-Q3-max)**
	5.04 (1.24)	4.55 (3.42-4.31-6.31-6.83)	5.15 (1.53)	4.96 (3.02-3.96-6.49-7.92)	5.13 (1.48)	4.75 (3.02-4.11-6.31-7.92)
**Response to left facial nerve stimulation at the same needle site (latency: msec)**						
No response		0 (0)		53 (24.3)		53 (19.2)

Not evaluated		8 (13.8)		6 (0.3)		14 (5.1)
Responsive		50 (86.2)		159 (72.9)		209 (75.7)
	**mean (SD.)**	**median (min-Q1-Q3-max)**	**mean (SD.)**	**median (min-Q1-Q3-max)**	**mean (SD.)**	**median (min-Q1-Q3-max)**
	3.33 (0.64)	3.15 (2.57-2.91-3.38-4.79)	4.73 (0.85)	4.16 (4.01-4.12-5.89-6.08)	4.4 (1)	4.13 (2.57-4.07-5.8-6.08)
**Response to left facial nerve stimulation at the same needle site (amplitude: μV)**						
No response						53 (19.2)
Not evaluated						14 (5.1)
Responsive						209 (75.7)
	**mean (SD.)**	**median (min-Q1-Q3-max)**	**mean (SD.)**	**median (min-Q1-Q3-max)**	**mean (SD.)**	**median (min-Q1-Q3-max)**
	5.34 (1.24)	4.63 (4.11-4.41-6.37-7.38)	4.27 (2.24)	5.16 (1-1.24-6.32-6.79)	4.53 (2.1)	5.16 (1-1.57-6.32-7.38)
**Response of upper left O.Oris to right facial nerve stimulation1 cm lateral (latency: msec)**						
No response		18 (31.03)		112 (51.38)		130 (47.1)
Responsive		40 (68.97)		106 (48.62)		146 (52.9)
	**mean (SD.)**	**median (min-Q1-Q3-max)**	**mean (SD.)**	**median (min-Q1-Q3-max)**	**mean (SD.)**	**median (min-Q1-Q3-max)**
	3.89 (0.37)	3.9 (3.41-3.55-4.23-4.39)	4.66 (2.16)	4.64 (2.27-2.52-6.82-6.99)	4.45 (1.88)	3.9 (2.27-2.57-6.76-6.99)
**Response of upper left O.Oris to right facial nerve stimulation1 cm lateral (amplitude: μV)**						
No response		18 (31.03)		112 (51.38)		130 (47.1)
Responsive		40 (68.97)		106 (48.62)		146 (52.9)
	**mean (SD.)**	**median (min-Q1-Q3-max)**	**mean (SD.)**	**median (min-Q1-Q3-max)**	**mean (SD.)**	**median (min-Q1-Q3-max)**
	5.34 (1.02)	5.35 (4.19-4.31-6.35-6.49)	4.9 (1.51)	4.88 (3.2-3.4-6.41-6.63)	5.02 (1.4)	5.3 (3.2-3.46-6.4-6.63)
**Response to left facial nerve stimulation at the same needle site (latency: msec)**						
No response		0 (0)		53 (24.3)		53 (19.2)
Not evaluated		18 (31)		112 (51.4)		130 (47.1)
Responsive		40 (69)		53 (24.3)		93 (33.7)

	**mean (SD.)**	**median (min-Q1-Q3-max)**	**mean (SD.)**	**median (min-Q1-Q3-max)**	**mean (SD.)**	**median (min-Q1-Q3-max)**
	3.03 (0.42)	3.05 (2.45-2.62-3.42-3.59)	4.1 (0.05)	4.11 (4.01-4.05-4.13-4.21)	3.64 (0.6)	4.03 (2.45-3.32-4.11-4.21)
**Response to left facial nerve stimulation at the same needle site (amplitude: μV)**						
No response		0 (0)				53 (19.2)
Not evaluated		18 (31)				130 (47.1)
Responsive		40 (69)				93 (33.7)
	**mean (SD.)**	**median (min-Q1-Q3-max)**	**mean (SD.)**	**median (min-Q1-Q3-max)**	**mean (SD.)**	**median (min-Q1-Q3-max)**
	5.93 (1.44)	5.94 (4.33-4.5-7.37-7.49)	4.19 (0.07)	4.18 (4.1-4.13-4.23-4.32)	4.94 (1.28)	4.28 (4.1-4.17-4.62-7.49)
**Response of upper left O.Oris to right facial nerve stimulation1,5 cm lateral (latency: msec)**						
No response		38 (65.52)		218 (100)		256 (92.8)
Responsive		20 (34.48)		0 (0)		20 (7.2)
	**mean (SD.)**	**median (min-Q1-Q3-max)**	**mean (SD.)**	**median (min-Q1-Q3-max)**	**mean (SD.)**	**median (min-Q1-Q3-max)**
	3.51 (0.06)	3.51 (3.41-3.46-3.56-3.61)	-	-	3.51 (0.06)	3.51 (3.41-3.46-3.56-3.61)
**Response of upper left O.Oris to right facial nerve stimulation1,5 cm lateral (amplitude: μV)**						
No response		38 (65.52)		218 (100)		256 (92.8)
Responsive		20 (34.48)		0 (0)		20 (7.2)
	**mean (SD.)**	**median (min-Q1-Q3-max)**	**mean (SD.)**	**median (min-Q1-Q3-max)**	**mean (SD.)**	**median (min-Q1-Q3-max)**
	4.42 (0.11)	4.38 (4.28-4.32-4.53-4.61)	-	-	4.42 (0.11)	4.38 (4.28-4.32-4.53-4.61)
**Response to left facial nerve stimulation at the same needle site (latency: msec)**						
Not evaluated		38 (65.52)		218 (100)		256 (92.8)
Responsive		20 (34.48)		0 (0)		20 (7.2)
	**mean (SD.)**	**median (min-Q1-Q3-max)**	**mean (SD.)**	**median (min-Q1-Q3-max)**	**mean (SD.)**	**median (min-Q1-Q3-max)**
	3.35 (0.12)	3.33 (3.18-3.23-3.49-3.51)	-	-	3.35 (0.12)	3.33 (3.18-3.23-3.49-3.51)
**Response to left facial nerve**						

**stimulation at the same needle site (latency: msec)**						
Not evaluated		38 (65.52)		218 (100)		256 (92.8)
Responsive		20 (34.48)		0 (0)		20 (7.2)
	**mean (SD.)**	**median (min-Q1-Q3-max)**	**mean (SD.)**	**median (min-Q1-Q3-max)**	**mean (SD.)**	**median (min-Q1-Q3-max)**
	4.61 (0.12)	4.62 (4.41-4.5-4.74-4.76)	-	-	4.61 (0.12)	4.62 (4.41-4.5-4.74-4.76)
**Response of upper left O.Oris to right facial nerve stimulation2 cm lateral (latency: msec)**						
No response		58 (100)		218 (100)		276 (100)
Responsive		0 (0)		0 (0)		0 (0)
**Response of upper left O.Oris to right facial nerve stimulation2 cm lateral (amplitude: µV)**						
No response		58 (100)		218 (100)		276 (100)
Responsive		0 (0)		0 (0)		0 (0)
**Response to left facial nerve stimulation at the same needle site (latency: msec)**						
Not evaluated		58 (100)		218 (100)		276 (100)
Responsive		0 (0)		0 (0)		0 (0)
**Response to left facial nerve stimulation at the same needle site (amplitude: µV)**						
Not evaluated		58 (100)		218 (100)		276 (100)
Responsive		0 (0)		0 (0)		0 (0)
**Response of upper left O.Oris to right facial nerve stimulation 2,5 cm lateral (latency: msec)**						
No response		58 (100)		218 (100)		276 (100)
Responsive		0 (0)		0 (0)		0 (0)
**Response of upper left O.Oris to right facial nerve**						

**stimulation 2,5 cm lateral (amplitude: μV)**						
No response	58 (100)		218 (100)		276 (100)	
Responsive	0 (0)		0 (0)		0 (0)	
**Response to left facial nerve stimulation at the same needle site (latency: msec)**						
Not evaluated	58 (100)		218 (100)		276 (100)	
Responsive	0 (0)		0 (0)		0 (0)	
**Response to left facial nerve stimulation at the same needle site (amplitude: μV)**						
Not evaluated	58 (100)		218 (100)		276 (100)	
Responsive	0 (0)		0 (0)		0 (0)	
**Response of upper right O.Oris to left facial nerve stimulation0,5 cm lateral (latency: msec)**						
No response	8 (13.79)		59 (27.06)		67 (24.3)	
Responsive	50 (86.21)		159 (72.94)		209 (75.7)	
	**mean (SD.)**	**median (min-Q1-Q3-max)**	**mean (SD.)**	**median (min-Q1-Q3-max)**	**mean (SD.)**	**median (min-Q1-Q3-max)**
	3.99 (0.44)	3.92 (3.31-3.63-4.19-4.95)	5.11 (0.99)	4.51 (4.15-4.38-6.47-6.59)	4.84 (1.01)	4.45 (3.31-4.3-6.3-6.59)
**Response of upper right O.Oris to left facial nerve stimulation 0,5 cm lateral (amplitude: μV)**						
No response	8 (13.79)		59 (27.06)		67 (24.3)	
Responsive	50 (86.21)		159 (72.94)		209 (75.7)	
	**mean (SD.)**	**median (min-Q1-Q3-max)**	**mean (SD.)**	**median (min-Q1-Q3-max)**	**mean (SD.)**	**median (min-Q1-Q3-max)**
	4.95 (1.1)	4.71 (3.07-4.11-6.14-6.64)	3.93 (2.07)	4.93 (0.55-1.31-5.56-6.52)	4.17 (1.93)	4.91 (0.55-1.77-5.6-6.64)
**Response to right facial nerve stimulation at the same needle site (latency: msec)**						
Not evaluated	8 (13.79)		6 (2.75)		14 (5.1)	
Responsive	50 (86.21)		212 (97.25)		262 (94.9)	

	**mean (SD.)**	**median (min-Q1-Q3-max)**	**mean (SD.)**	**median (min-Q1-Q3-max)**	**mean (SD.)**	**median (min-Q1-Q3-max)**
	3.47 (0.58)	3.32 (2.78-3.09-3.55-4.67)	4.04 (1.01)	4.53 (2.19-3.32-4.68-4.94)	3.93 (0.97)	4.49 (2.19-3.09-4.65-4.94)
**Response to right facial nerve stimulation at the same needle site (amplitude: μV)**						
Not evaluated		8 (13.79)		6 (2.75)		14 (5.1)
Responsive		50 (86.21)		212 (97.25)		262 (94.9)
	**mean (SD.)**	**median (min-Q1-Q3-max)**	**mean (SD.)**	**median (min-Q1-Q3-max)**	**mean (SD.)**	**median (min-Q1-Q3-max)**
	5.13 (1.03)	4.83 (3.52-4.45-6.25-6.55)	5.31 (0.93)	5 (4.03-4.5-5.99-6.99)	5.28 (0.95)	4.96 (3.52-4.49-6.22-6.99)
**Response of upper right O.Oris to left facial nerve stimulation1 cm lateral (latency: msec)**						
No response		8 (13.79)		112 (51.38)		120 (43.5)
Responsive		50 (86.21)		106 (48.62)		156 (56.5)
	**mean (SD.)**	**median (min-Q1-Q3-max)**	**mean (SD.)**	**median (min-Q1-Q3-max)**	**mean (SD.)**	**median (min-Q1-Q3-max)**
	3.98 (0.49)	4.11 (3.11-3.57-4.31-4.82)	5.83 (1.45)	5.82 (4.22-4.4-7.27-7.4)	5.24 (1.5)	4.45 (3.11-4.26-7.22-7.4)
**Response of upper right O.Oris to left facial nerve stimulation 1 cm lateral (amplitude: μV)**						
No response		8 (13.79)		112 (51.38)		120 (43.5)
Responsive		50 (86.21)		106 (48.62)		156 (56.5)
	**mean (SD.)**	**median (min-Q1-Q3-max)**	**mean (SD.)**	**median (min-Q1-Q3-max)**	**mean (SD.)**	**median (min-Q1-Q3-max)**
	4.98 (1.01)	4.69 (3.25-4.42-6.05-6.55)	2.84 (2.29)	2.5 (0.22-0.59-5.17-5.97)	3.52 (2.21)	4.45 (0.22-0.79-5.45-6.55)
**Response to right facial nerve stimulation at the same needle site (latency: msec)**						
Not evaluated		8 (13.79)		59 (27.06)		67 (24.3)
Responsive		50 (86.21)		159 (72.94)		209 (75.7)
	**mean (SD.)**	**median (min-Q1-Q3-max)**	**mean (SD.)**	**median (min-Q1-Q3-max)**	**mean (SD.)**	**median (min-Q1-Q3-max)**
	3.44 (0.7)	3.41 (2.52-2.78-3.77-4.76)	3.72 (1.02)	4.32 (2.07-2.38-4.51-4.75)	3.66 (0.96)	4.21 (2.07-2.52-4.5-4.76)
**Response to right facial**						

**nerve stimulation at the same needle site (amplitude: µV)**						
Not evaluated		8 (13.79)		59 (27.06)		67 (24.3)
Responsive		50 (86.21)		159 (72.94)		209 (75.7)
	**mean (SD.)**	**median (min-Q1-Q3-max)**	**mean (SD.)**	**median (min-Q1-Q3-max)**	**mean (SD.)**	**median (min-Q1-Q3-max)**
	5.1 (1.03)	4.74 (3.46-4.36-6.19-6.65)	5.14 (0.92)	4.69 (4.01-4.45-6.11-6.97)	5.13 (0.95)	4.71 (3.46-4.41-6.15-6.97)
**Response of upper right O.Oris to left facial nerve stimulation1,5 cm lateral (latency: msec)**						
No response						203 (73.6)
Responsive		38 (65.52)		165 (75.69)		73 (26.4)
		20 (34.48)		53 (24.31)		
	**mean (SD.)**	**median (min-Q1-Q3-max)**	**mean (SD.)**	**median (min-Q1-Q3-max)**	**mean (SD.)**	**median (min-Q1-Q3-max)**
	4.03 (0.14)	4.03 (3.81-3.92-4.14-4.28)	4.75 (0.23)	4.78 (4.29-4.61-4.87-5.38)	4.55 (0.38)	4.65 (3.81-4.25-4.83-5.38)
**Response of upper right O.Oris to left facial nerve stimulation1,5cm lateral (amplitude: µV)**						
No response		38 (65.52)		165 (75.69)		203 (73.6)
Responsive		20 (34.48)		53 (24.31)		73 (26.4)
	**mean (SD.)**	**median (min-Q1-Q3-max)**	**mean (SD.)**	**median (min-Q1-Q3-max)**	**mean (SD.)**	**median (min-Q1-Q3-max)**
	5.01 (0.8)	5.02 (3.73-4.42-5.69-6.25)	5.22 (0.2)	5.21 (4.86-5.09-5.39-5.65)	5.17 (0.45)	5.21 (3.73-5.02-5.41-6.25)
**Response to right facial nerve stimulation at the same needle site (latency: msec)**						
Not evaluated		38 (65.52)		112 (51.38)		150 (54.3)
Responsive		20 (34.48)		106 (48.62)		126 (45.7)
	**mean (SD.)**	**median (min-Q1-Q3-max)**	**mean (SD.)**	**median (min-Q1-Q3-max)**	**mean (SD.)**	**median (min-Q1-Q3-max)**
	2.7 (0.17)	2.7 (2.35-2.6-2.8-2.99)	3.48 (1.22)	3.46 (2.01-2.22-4.71-4.97)	3.36 (1.16)	2.71 (2.01-2.28-4.66-4.97)
**Response to right facial nerve stimulation at the same needle site (amplitude: µV)**						

Not evaluated		38 (65.52)		112 (51.38)		150 (54.3)
Responsive		20 (34.48)		106 (48.62)		126 (45.7)
	**mean (SD.)**	**median (min-Q1-Q3-max)**	**mean (SD.)**	**median (min-Q1-Q3-max)**	**mean (SD.)**	**median (min-Q1-Q3-max)**
	5.22 (0.71)	5.27 (3.78-4.67-5.77-6.38)	5.1 (1.1)	5.25 (3.09-4.05-6.15-6.75)	5.12 (1.04)	5.25 (3.09-4.27-6.1-6.75)
**Response of upper right O.Oris to left facial nerve stimulation2 cm lateral (latency: msec)**						
No response		58 (100)		218 (100)		276 (100)
Responsive		0 (0)		0 (0)		0 (0)
**Response of upper right O.Oris to left facial nerve stimulation2cm lateral (amplitude: µV)**						
No response		58 (100)		218 (100)		276 (100)
Responsive		0 (0)		0 (0)		0 (0)
**Response to right facial nerve stimulation at the same needle site (latency: msec)**						
Not evaluated		58 (100)		165 (75.69)		223 (80.8)
Responsive		0 (0)		53 (24.31)		53 (19.2)
	**mean (SD.)**	**median (min-Q1-Q3-max)**	**mean (SD.)**	**median (min-Q1-Q3-max)**	**mean (SD.)**	**median (min-Q1-Q3-max)**
	-	-	2.26 (0.14)	2.27 (1.95-2.17-2.36-2.57)	2.26 (0.14)	2.27 (1.95-2.17-2.36-2.57)
**Response to right facial nerve stimulation at the same needle site (amplitude: µV)**						
Not evaluated		58 (100)		165 (75.69)		223 (80.8)
Responsive		0 (0)		53 (24.31)		53 (19.2)
	**mean (SD.)**	**median (min-Q1-Q3-max)**	**mean (SD.)**	**median (min-Q1-Q3-max)**	**mean (SD.)**	**median (min-Q1-Q3-max)**
	-	-	4.44 (0.55)	4.38 (3.57-3.93-4.81-6.12)	4.44 (0.55)	4.38 (3.57-3.93-4.81-6.12)
**Response of upper right O.Oris to left facial nerve stimulation2,5 cm lateral (latency: msec)**						

No response		58 (100)		218 (100)		276 (100)
Responsive		0 (0)		0 (0)		0 (0)
**Response of upper right O.Oris to left facial nerve stimulation2,5cm lateral (amplitude: μV)**						
No response		58 (100)		218 (100)		276 (100)
Responsive		0 (0)		0 (0)		0 (0)
**Response to right facial nerve stimulation at the same needle site (latency: msec)**						
Not evaluated		58 (100)		165 (75.69)		223 (80.8)
Responsive		0 (0)		53 (24.31)		53 (19.2)
	**mean (SD.)**	**median (min-Q1-Q3-max)**	**mean (SD.)**	**median (min-Q1-Q3-max)**	**mean (SD.)**	**median (min-Q1-Q3-max)**
	0 (0)	0 (0-0-0-0)	2.27 (0.14)	2.27 (1.94-2.18-2.37-2.56)	2.27 (0.14)	2.27 (1.94-2.18-2.37-2.56)
**Response to right facial nerve stimulation at the same needle site (amplitude: μV)**						
Not evaluated		58 (100)		165 (75.69)		223 (80.8)
Responsive		0 (0)		53 (24.31)		53 (19.2)
	**mean (SD.)**	**median (min-Q1-Q3-max)**	**mean (SD.)**	**median (min-Q1-Q3-max)**	**mean (SD.)**	**median (min-Q1-Q3-max)**
	0 (0)	0 (0-0-0-0)	4.26 (0.44)	4.25 (3.55-3.91-4.56-5.25)	4.26 (0.44)	4.25 (3.55-3.91-4.56-5.25)
**Response of lower left O.Oris to right facial nerve stimulation0,5 cm lateral (latency: msec)**						
No response		8 (13.79)		6 (2.75)		14 (5.1)
Responsive		50 (86.21)		212 (97.25)		262 (94.9)
	**mean (SD.)**	**median (min-Q1-Q3-max)**	**mean (SD.)**	**median (min-Q1-Q3-max)**	**mean (SD.)**	**median (min-Q1-Q3-max)**
	4.05 (0.63)	4.06 (3.05-3.5-4.35-5.25)	4.81 (1.11)	4.7 (3.01-4.06-5.65-7.21)	4.66 (1.08)	4.56 (3.01-3.82-5.39-7.21)
**Response of lower left O.Oris to right facial nerve stimulation0,5 cm lateral (amplitude: μV)**						

No response		8 (13.79)		6 (2.75)		14 (5.1)
Responsive		50 (86.21)		212 (97.25)		262 (94.9)
	**mean (SD.)**	**median (min-Q1-Q3-max)**	**mean (SD.)**	**median (min-Q1-Q3-max)**	**mean (SD.)**	**median (min-Q1-Q3-max)**
	4.78 (0.93)	4.41 (3.25-4.09-5.78-6.31)	4.63 (0.86)	4.64 (3.09-3.99-5.05-8.81)	4.65 (0.87)	4.61 (3.09-4.07-5.16-8.81)
**Response to left facial nerve stimulation at the same needle site (latency: msec)**						
Not evaluated		8 (13.79)		6 (2.75)		67 (24.3)
Responsive		50 (86.21)		212 (97.25)		209 (75.7)
	**mean (SD.)**	**median (min-Q1-Q3-max)**	**mean (SD.)**	**median (min-Q1-Q3-max)**	**mean (SD.)**	**median (min-Q1-Q3-max)**
	3.99 (0.72)	3.95 (3.02-3.32-4.45-5.39)	5.13 (1.07)	4.51 (4.02-4.31-6.25-6.99)	4.86 (1.11)	4.42 (3.02-4.22-6.05-6.99)
**Response to left facial nerve stimulation at the same needle site (amplitude: µV)**						
Not evaluated		8 (13.79)		59 (27.06)		67 (24.3)
Responsive		50 (86.21)		159 (72.94)		209 (75.7)
	**mean (SD.)**	**median (min-Q1-Q3-max)**	**mean (SD.)**	**median (min-Q1-Q3-max)**	**mean (SD.)**	**median (min-Q1-Q3-max)**
	5 (1.01)	4.63 (3.19-4.27-6.08-6.53)	3.3 (1.67)	3.81 (0.48-1.45-4.8-5.78)	3.71 (1.7)	4.18 (0.48-1.83-4.91-6.53)
**Response of lower left O.Oris to right facial nerve stimulation1 cm lateral (latency: msec)**						
No response		8 (13.79)		59 (27.06)		183 (66.3)
Responsive		50 (86.21)		159 (72.94)		93 (33.7)
	**mean (SD.)**	**median (min-Q1-Q3-max)**	**mean (SD.)**	**median (min-Q1-Q3-max)**	**mean (SD.)**	**median (min-Q1-Q3-max)**
	3.83 (0.37)	3.84 (3.24-3.5-4.17-4.41)	5.53 (0.5)	5.46 (4.58-5.19-5.88-6.48)	4.8 (0.96)	4.91 (3.24-4.05-5.56-6.48)
**Response of lower left O.Oris to right facial nerve stimulation1 cm lateral (amplitude: µV)**						
No response		18 (31.03)		165 (75.69)		183 (66.3)
Responsive		40 (68.97)		53 (24.31)		93 (33.7)
	**mean (SD.)**	**median (min-Q1-Q3-max)**	**mean (SD.)**	**median (min-Q1-Q3-max)**	**mean (SD.)**	**median (min-Q1-Q3-max)**
	5.14 (0.88)	5.09 (3.81-4.32-6-6.38)	4.07 (0.42)	4.01 (3.28-3.71-4.43-4.91)	4.53 (0.84)	4.32 (3.28-3.91-4.71-6.38)

**Response to left facial nerve stimulation at the same needle site (latency: msec)**						
Not evaluated		18 (31.03)		165 (75.69)		183 (66.3)
Responsive		40 (68.97)		53 (24.31)		93 (33.7)
	**mean (SD.)**	**median (min-Q1-Q3-max)**	**mean (SD.)**	**median (min-Q1-Q3-max)**	**mean (SD.)**	**median (min-Q1-Q3-max)**
	3.77 (0.3)	3.75 (3.35-3.5-3.99-4.55)	2.99 (0.27)	3.06 (2.41-2.79-3.19-3.48)	3.33 (0.48)	3.28 (2.41-2.95-3.67-4.55)
**Response to left facial nerve stimulation at the same needle site (amplitude: µV)**						
Not evaluated		18 (31.03)		165 (75.69)		183 (66.3)
Responsive		40 (68.97)		53 (24.31)		93 (33.7)
	**mean (SD.)**	**median (min-Q1-Q3-max)**	**mean (SD.)**	**median (min-Q1-Q3-max)**	**mean (SD.)**	**median (min-Q1-Q3-max)**
	5.34 (0.87)	5.15 (4.16-4.51-6.22-6.55)	4.79 (0.72)	4.94 (3.28-4.19-5.33-6.02)	5.03 (0.83)	4.94 (3.28-4.41-5.78-6.55)
**Response of lower left O.Oris to right facial nerve stimulation1,5 cm lateral (latency: msec)**						
No response		38 (65.52)		165 (75.69)		203 (73.6)
Responsive		20 (34.48)		53 (24.31)		73 (26.4)
	**mean (SD.)**	**median (min-Q1-Q3-max)**	**mean (SD.)**	**median (min-Q1-Q3-max)**	**mean (SD.)**	**median (min-Q1-Q3-max)**
	3.56 (0.2)	3.58 (3.21-3.4-3.69-3.89)	5.31 (0.45)	5.27 (4.49-5.02-5.57-6.29)	4.83 (0.88)	5.06 (3.21-3.85-5.43-6.29)
**Response of lower left O.Oris to right facial nerve stimulation1,5 cm lateral (amplitude: µV)**						
No response		38 (65.52)		165 (75.69)		203 (73.6)
Responsive		20 (34.48)		53 (24.31)		73 (26.4)
	**mean (SD.)**	**median (min-Q1-Q3-max)**	**mean (SD.)**	**median (min-Q1-Q3-max)**	**mean (SD.)**	**median (min-Q1-Q3-max)**
	4.09 (0.52)	4.13 (3.15-3.7-4.57-4.74)	4.2 (0.53)	4.31 (3.09-3.79-4.59-4.99)	4.17 (0.53)	4.31 (3.09-3.76-4.59-4.99)
**Response to left facial nerve stimulation at the same needle site (latency: msec)**						

Not evaluated		38 (65.52)		165 (75.69)		203 (73.6)
Responsive		20 (34.48)		53 (24.31)		73 (26.4)
	**mean (SD.)**	**median (min-Q1-Q3-max)**	**mean (SD.)**	**median (min-Q1-Q3-max)**	**mean (SD.)**	**median (min-Q1-Q3-max)**
	3.37 (0.16)	3.36 (3.11-3.26-3.47-3.69)	9.4 (46.43)	3.09 (2.41-2.75-3.32-341)	7.75 (39.55)	3.21 (2.41-2.85-3.37-341)
**Response to left facial nerve stimulation at the same needle site (amplitude: μV)**						
Not evaluated		38 (65.52)		165 (75.69)		203 (73.6)
Responsive		20 (34.48)		53 (24.31)		73 (26.4)
	**mean (SD.)**	**median (min-Q1-Q3-max)**	**mean (SD.)**	**median (min-Q1-Q3-max)**	**mean (SD.)**	**median (min-Q1-Q3-max)**
	4.3 (0.37)	4.29 (3.64-4.06-4.6-4.89)	3.17 (0.85)	2.93 (2.11-2.52-3.52-5.29)	3.48 (0.9)	3.39 (2.11-2.65-4.29-5.29)
**Response of lower left O.Oris to right facial nerve stimulation2cm lateral (latency: msec)**						
No response		58 (100)		218 (100)		276 (100)
Responsive		0 (0)		0 (0)		0 (0)
**Response of lower left O.Oris to right facial nerve stimulation2 cm lateral (amplitude: μV)**						
No response		58 (100)		218 (100)		276 (100)
Responsive		0 (0)		0 (0)		0 (0)
**Response to left facial nerve stimulation at the same needle site (latency: msec)**						
Not evaluated		58 (100)		218 (100)		276 (100)
Responsive		0 (0)		0 (0)		0 (0)
**Response to left facial nerve stimulation at the same needle site (amplitude: μV)**						
Not evaluated		58 (100)		218 (100)		276 (100)
Responsive		0 (0)		0 (0)		0 (0)
**Response of lower left**						

**O.Oris to right facial nerve stimulation2,5cm lateral (latency: msec)**						
No response	58 (100)		218 (100)		276 (100)	
Responsive	0 (0)		0 (0)		0 (0)	
**Response of lower left O.Oris to right facial nerve stimulation2,5 cm lateral (amplitude: μV)**						
No response	58 (100)		218 (100)		276 (100)	
Responsive	0 (0)		0 (0)		0 (0)	
**Response to left facial nerve stimulation at the same needle site (latency: msec)**						
Not evaluated	58 (100)		218 (100)		276 (100)	
Responsive	0 (0)		0 (0)		0 (0)	
**Response to left facial nerve stimulation at the same needle site (amplitude: μV)**						
Not evaluated	58 (100)		218 (100)		276 (100)	
Responsive	0 (0)		0 (0)		0 (0)	
**Response of lower right O.Oris to left facial nerve stimulation0,5cm lateral (latency: msec)**						
No response	8 (13.79)		59 (27.06)		67 (24.3)	
Responsive	50 (86.21)		159 (72.94)		209 (75.7)	
	**mean (SD.)**	**median (min-Q1-Q3-max)**	**mean (SD.)**	**median (min-Q1-Q3-max)**	**mean (SD.)**	**median (min-Q1-Q3-max)**
	4.01 (0.51)	4.1 (3.17-3.52-4.29-4.89)	4.94 (0.7)	4.71 (4.02-4.47-5.33-7.35)	4.72 (0.77)	4.58 (3.17-4.26-5.17-7.35)
**Response of lower right O.Oris to left facial nerve stimulation0,5 cm lateral (amplitude: μV)**						
No response	8 (13.79)		59 (27.06)		67 (24.3)	
Responsive	50 (86.21)		159 (72.94)		209 (75.7)	

	**mean (SD.)**	**median (min-Q1-Q3-max)**	**mean (SD.)**	**median (min-Q1-Q3-max)**	**mean (SD.)**	**median (min-Q1-Q3-max)**
	4.71 (0.76)	4.66 (3.19-4.23-5.18-5.98)	3.74 (2.03)	4.67 (0.37-1.27-5.38-6.02)	3.97 (1.86)	4.67 (0.37-1.92-5.36-6.02)
**Response to right facial nerve stimulation at the same needle site (latency: msec)**						
Not evaluated		8 (13.79)		6 (2.75)		14 (5.1)
Responsive		50 (86.21)		212 (97.25)		262 (94.9)
	**mean (SD.)**	**median (min-Q1-Q3-max)**	**mean (SD.)**	**median (min-Q1-Q3-max)**	**mean (SD.)**	**median (min-Q1-Q3-max)**
	3.87 (0.53)	3.79 (3.09-3.42-4.29-4.89)	4.04 (0.96)	4.29 (2.12-3.3-4.85-5.21)	4 (0.89)	4.21 (2.12-3.42-4.72-5.21)
**Response to right facial nerve stimulation at the same needle site (amplitude: μV)**						
Not evaluated		8 (13.79)		6 (2.75)		14 (5.1)
Responsive		50 (86.21)		212 (97.25)		262 (94.9)
	**mean (SD.)**	**median (min-Q1-Q3-max)**	**mean (SD.)**	**median (min-Q1-Q3-max)**	**mean (SD.)**	**median (min-Q1-Q3-max)**
	4.97 (0.85)	4.8 (3.45-4.46-5.91-6.27)	4.46 (0.67)	4.42 (3.06-4.11-4.9-5.98)	4.55 (0.73)	4.49 (3.06-4.13-4.95-6.27)
**Response of lower right O.Oris to left facial nerve stimulation1cm lateral (latency: msec)**						
No response		28 (48.28)		165 (75.69)		193 (69.9)
Responsive		30 (51.72)		53 (24.31)		83 (30.1)
	**mean (SD.)**	**median (min-Q1-Q3-max)**	**mean (SD.)**	**median (min-Q1-Q3-max)**	**mean (SD.)**	**median (min-Q1-Q3-max)**
	4.03 (0.64)	3.72 (3.38-3.55-4.71-5.15)	4.09 (0.19)	4.09 (3.75-3.93-4.22-4.49)	4.07 (0.41)	4.02 (3.38-3.79-4.25-5.15)
**Response of lower right O.Oris to left facial nerve stimulation1 cm lateral (amplitude: μV)**						
No response		28 (48.28)		165 (75.69)		193 (69.9)
Responsive		30 (51.72)		53 (24.31)		83 (30.1)
	**mean (SD.)**	**median (min-Q1-Q3-max)**	**mean (SD.)**	**median (min-Q1-Q3-max)**	**mean (SD.)**	**median (min-Q1-Q3-max)**
	4.31 (0.43)	4.47 (3.51-3.91-4.64-4.91)	4.37 (0.42)	4.39 (3.67-4.06-4.69-5.11)	4.35 (0.42)	4.41 (3.51-3.98-4.69-5.11)
**Response to right facial**						

**nerve stimulation at the same needle site (latency: msec)**						
Not evaluated		28 (48.28)		112 (51.38)		140 (50.7)
Responsive		30 (51.72)		106 (48.62)		136 (49.3)
	**mean (SD.)**	**median (min-Q1-Q3-max)**	**mean (SD.)**	**median (min-Q1-Q3-max)**	**mean (SD.)**	**median (min-Q1-Q3-max)**
	3.93 (0.66)	3.56 (3.23-3.46-4.73-4.99)	3.3 (0.94)	3.07 (2.01-2.46-4.3-4.89)	3.44 (0.93)	3.45 (2.01-2.59-4.43-4.99)
**Response to right facial nerve stimulation at the same needle site (amplitude: μV)**						
Not evaluated		58 (100)		218 (100)		276 (100)
Responsive		0 (0)		0 (0)		0 (0)
**Response of lower right O.Oris to left facial nerve stimulation1,5cm lateral (latency: msec)**						
No response		58 (100)		218 (100)		276 (100)
Responsive		0 (0)		0 (0)		0 (0)
**Response of lower right O.Oris to left facial nerve stimulation1,5 cm lateral (amplitude: μV)**						
No response		58 (100)		218 (100)		276 (100)
Responsive		0 (0)		0 (0)		0 (0)
**Response to right facial nerve stimulation at the same needle site (latency: msec)**						
Not evaluated		58 (100)		165 (75.69)		223 (80.8)
Responsive		0 (0)		53 (24.31)		53 (19.2)
	**mean (SD.)**	**median (min-Q1-Q3-max)**	**mean (SD.)**	**median (min-Q1-Q3-max)**	**mean (SD.)**	**median (min-Q1-Q3-max)**
	-	-	2.04 (0.19)	2.05 (1.7-1.89-2.19-2.42)	2.04 (0.19)	2.05 (1.7-1.89-2.19-2.42)
**Response to right facial nerve stimulation at the same needle site (amplitude: μV)**						

Not evaluated	58 (100)		165 (75.69)		223 (80.8)	
Responsive	0 (0)		53 (24.31)		53 (19.2)	
	**mean (SD.)**	**median (min-Q1-Q3-max)**	**mean (SD.)**	**median (min-Q1-Q3-max)**	**mean (SD.)**	**median (min-Q1-Q3-max)**
	-	-	3.7 (0.7)	3.69 (2.41-3.25-4.19-4.98)	3.7 (0.7)	3.69 (2.41-3.25-4.19-4.98)
**Response of lower right O.Oris to left facial nerve stimulation2cm lateral (latency: msec)**						
No response	58 (100)		218 (100)		276 (100)	
Responsive	0 (0)		0 (0)		0 (0)	
**Response of lower right O.Oris to left facial nerve stimulation2cm lateral (amplitude: μV)**						
No response	58 (100)		218 (100)		276 (100)	
Responsive	0 (0)		0 (0)		0 (0)	
**Response to right facial nerve stimulation at the same needle site (latency: msec)**						
Not evaluated	58 (100)		165 (75.69)		223 (80.8)	
Responsive	0 (0)		53 (24.31)		53 (19.2)	
	**mean (SD.)**	**median (min-Q1-Q3-max)**	**mean (SD.)**	**median (min-Q1-Q3-max)**	**mean (SD.)**	**median (min-Q1-Q3-max)**
	-	-	2.33 (0.36)	2.27 (1.8-2.03-2.55-3.15)	2.33 (0.36)	2.27 (1.8-2.03-2.55-3.15)
**Response to right facial nerve stimulation at the same needle site (amplitude: μV)**						
Not evaluated	58 (100)		165 (75.69)		223 (80.8)	
Responsive	0 (0)		53 (24.31)		53 (19.2)	
	**mean (SD.)**	**median (min-Q1-Q3-max)**	**mean (SD.)**	**median (min-Q1-Q3-max)**	**mean (SD.)**	**median (min-Q1-Q3-max)**
	-	-	4.06 (0.7)	4.13 (2.63-3.63-4.58-5.29)	4.06 (0.7)	4.13 (2.63-3.63-4.58-5.29)
**Response of lower right O.Oris to left facial nerve stimulation2,5cm lateral (latency: msec)**						

No response		58 (100)		218 (100)		276 (100)
Responsive		0 (0)		0 (0)		0 (0)
**Response of lower right O.Oris to left facial nerve stimulation2,5cm lateral (amplitude: μV)**						
No response		58 (100)		218 (100)		276 (100)
Responsive		0 (0)		0 (0)		0 (0)
**Response to right facial nerve stimulation at the same needle site (latency: msec)**						
Not evaluated		58 (100)		165 (75.69)		223 (80.8)
Responsive		0 (0)		53 (24.31)		53 (19.2)
	**mean (SD.)**	**median (min-Q1-Q3-max)**	**mean (SD.)**	**median (min-Q1-Q3-max)**	**mean (SD.)**	**median (min-Q1-Q3-max)**
	-	-	2.27 (0.24)	2.29 (1.81-2.07-2.45-2.71)	2.27 (0.24)	2.29 (1.81-2.07-2.45-2.71)
**Response to right facial nerve stimulation at the same needle site (amplitude: μV)**						
Not evaluated		58 (100)		165 (75.69)		223 (80.8)
Responsive		0 (0)		53 (24.31)		53 (19.2)
	**mean (SD.)**	**median (min-Q1-Q3-max)**	**mean (SD.)**	**median (min-Q1-Q3-max)**	**mean (SD.)**	**median (min-Q1-Q3-max)**
	-	-	4.11 (0.63)	4.23 (2.93-3.58-4.66-5.06)	4.11 (0.63)	4.23 (2.93-3.58-4.66-5.06)

SD.:Standard deviation, min:minimum, Q1: Percentile 25,Q3: Percentile 75, max: Maximum PFP:peripheral facial palsy msec:millisecond μV:microvolt

Table S4. Denervation, reinnervation, recruitment and interference pattern at all needle insertion sites.

	Controls (n = 58)	PFP (n = 218)	Total (n = 276)
		n (%)	
**Denervation potential**			
Absent	58 (100)	112 (51.4)	170 (61.6)
Present	0 (0)	106 (48.6)	106 (38.4)
**Regeneration motor unit potential**			
Absent	58 (100)	106 (48.6)	164 (59.4)
Present	0 (0)	112 (51.4)	112 (40.6)
**Chronic neurogenic motor unit potential**			
Absent	58 (100)	106 (48.6)	164 (59.4)
Present	0 (0)	112 (51.4)	112 (40.6)
**Recruitment pattern**			
Decreased	0 (0)	112 (51.4)	112 (40.6)
Normal	58 (100)	53 (24.3)	111 (40.2)
Absent	0 (0)	53 (24.3)	53 (19.2)
**Interference pattern**			

Pattern	0 (0)	110 (50.5)	110 (39.9)
Exact	58 (100)	53 (24.3)	111 (40.2)
One oscillation	0 (0)	2 (0.9)	2 (0.7)
Absent	0 (0)	53 (24.3)	53 (19.2)

PFP:peripheral facial palsy.
